# Methylation‐ and homologous recombination deficiency‐related mutant genes predict the prognosis of lung adenocarcinoma

**DOI:** 10.1002/jcla.24277

**Published:** 2022-03-03

**Authors:** Guang‐Jie Nie, Jian Liu, Ai‐Mei Zou, Shao‐Feng Zhan, Jia‐Kang Liang, Yi Sui, Yu‐Ning Chen, Wei‐Shen Yao

**Affiliations:** ^1^ Department of Thoracic Surgery Shunde Hospital of Southern Medical University (The First People's Hospital of Shunde, Foshan, Guangdong, China) Foshan China; ^2^ Department of Pulmonary and Critical Care Medicine First People's Hospital of Foshan Affiliated Hospital of Sun Yat‐sen University in Foshan Foshan China; ^3^ Department of Oncology Shunde Hospital of Southern Medical University (The First People's Hospital of Shunde, Foshan, Guangdong, China) Foshan China; ^4^ Department of Oncology The First Affiliated Hospital of Guangzhou University of Traditional Chinese Medicine Guangzhou China; ^5^ Department of IVD Medical Marketing 3D Medicine Inc. Shanghai China; ^6^ Department of Surgery ShunDe Hospital Guangzhou University of Chinese Medicine Foshan Guangdong China; ^7^ Department of Thoracic Surgery Nanhai District People's Hospital Foshan China

**Keywords:** homologous recombination deficiency, lung adenocarcinoma, methylation, prognosis

## Abstract

**Background:**

Lung adenocarcinoma (LUAD) is a lung cancer subtype with poor prognosis. We investigated the prognostic value of methylation‐ and homologous recombination deficiency (HRD)‐associated gene signatures in LUAD.

**Methods:**

Data on RNA sequencing, somatic mutations, and methylation were obtained from TCGA database. HRD scores were used to stratify patients with LUAD into high and low HRD groups and identify differentially mutated and expressed genes (DMEGs). Pearson correlation analysis between DMEGs and methylation yielded methylation‐associated DMEGs. Cox regression analysis was used to construct a prognostic model, and the distribution of clinical features in the high‐ and low‐risk groups was compared.

**Results:**

Patients with different HRD scores showed different DNA mutation patterns. There were 272 differentially mutated genes and 6294 differentially expressed genes. Fifty‐seven DMEGs were obtained; the top 10 upregulated genes were *COL11A1*, *EXO1*, *ASPM*, *COL12A1*, *COL2A1*, *COL3A1*, *COL5A2*, *DIAPH3*, *CAD*, and *SLC25A13*, while the top 10 downregulated genes were *C7*, *ERN2*, *DLC1*, *SCN7A*, *SMARCA2*, *CARD11*, *LAMA2*, *ITIH5*, *FRY*, and *EPHB6*. Forty‐two DMEGs were negatively correlated with 259 methylation sites. Gene ontology and pathway enrichment analysis of the DMEGs revealed enrichment of loci involved in extracellular matrix‐related remodeling and signaling. Six out of the 42 methylation‐associated DMEGs were significantly associated with LUAD prognosis and included in the prognostic model. The model effectively stratified high‐ and low‐risk patients, with the high‐risk group having more patients with advanced stage disease.

**Conclusion:**

We developed a novel prognostic model for LUAD based on methylation and HRD. Methylation‐associated DMEGs may function as biomarkers and therapeutic targets for LUAD. Further studies are needed to elucidate their roles in LUAD carcinogenesis.

## Graphical abstract

This study aimed to investigat the prognostic value of methylation‐ and homologous recombination deficiency (HRD)‐associated gene signatures in lung adenocarcinoma (LUAD). A novel prognostic model for LUAD was developed based on methylation and HRD. Methylation‐associated DMEGs may function as both biomarkers and therapeutic targets for LUAD, and further studies are planned to elucidate their roles in the carcinogenesis of LUAD.

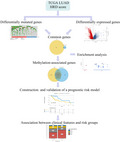

## 1 INTRODUCTION

Lung cancer is one of the most common cancers worldwide and has one of the highest mortality rates.^1^ Lung adenocarcinoma (LUAD) is a major subtype of lung cancer, accounting for 40% of lung cancers and has an overall 5‐year survival rate of less than 20%.^2^, ^3^, ^4^ Although the clinical outcomes of patients with LUAD have improved, treatment failure is common and occurs as a result of unresponsiveness or resistance to treatment, tumor recurrence, and metastasis.

Cancers, including LUAD, have high heterogeneity, which is a critical determinant of treatment success and outcomes. Molecular classification has shown a great potential in both distinguishing internal heterogeneity and stratifying LUAD into different subtypes.^4^, ^5^, ^6^, ^7^ Several studies have shown that some molecular classifications can predict the prognosis of LUAD.^4^, ^8^, ^9^, ^10^, ^11^

Homologous recombination repair is a conserved process which ensures that DNA is replicated correctly to avoid detrimental mutation‐induced damage.^12^ However, homologous recombination deficiency (HRD) is common in cancers, and this deficiency leads to impaired DNA repair and drives malignancy.^13^, ^14^ Recent studies have reported HRD in lung cancers^15^, ^16^; Diossy et al. identified a subset of LUAD with HRD, without the loss of the key homologous recombination genes *BRCA1*/*2*.^15^ Some cancers with HRD showed enhanced responses to poly (ADP‐ribose) polymerase inhibitors and platinum‐based chemotherapies; therefore, HRD may serve as a biomarker for the response to these drugs.^16^, ^17^, ^18^ Although HRD showed prognostic value for ovarian cancer,^19^ its efficacy in predicting the prognosis of patients with LUAD remains unexplored.

DNA methylation plays a critical role in regulating gene expression and is frequently dysregulated in cancers.^20^, ^21^, ^22^, ^23^ Thus, DNA methylation is a biomarker for cancers, and its signatures can predict survival in multiple cancers, including LUAD.^24^, ^25^, ^26^, ^27^, ^28^ However, whether methylation‐associated mutated genes also possess prognostic value for LUAD remains unknown.

**FIGURE 1:**
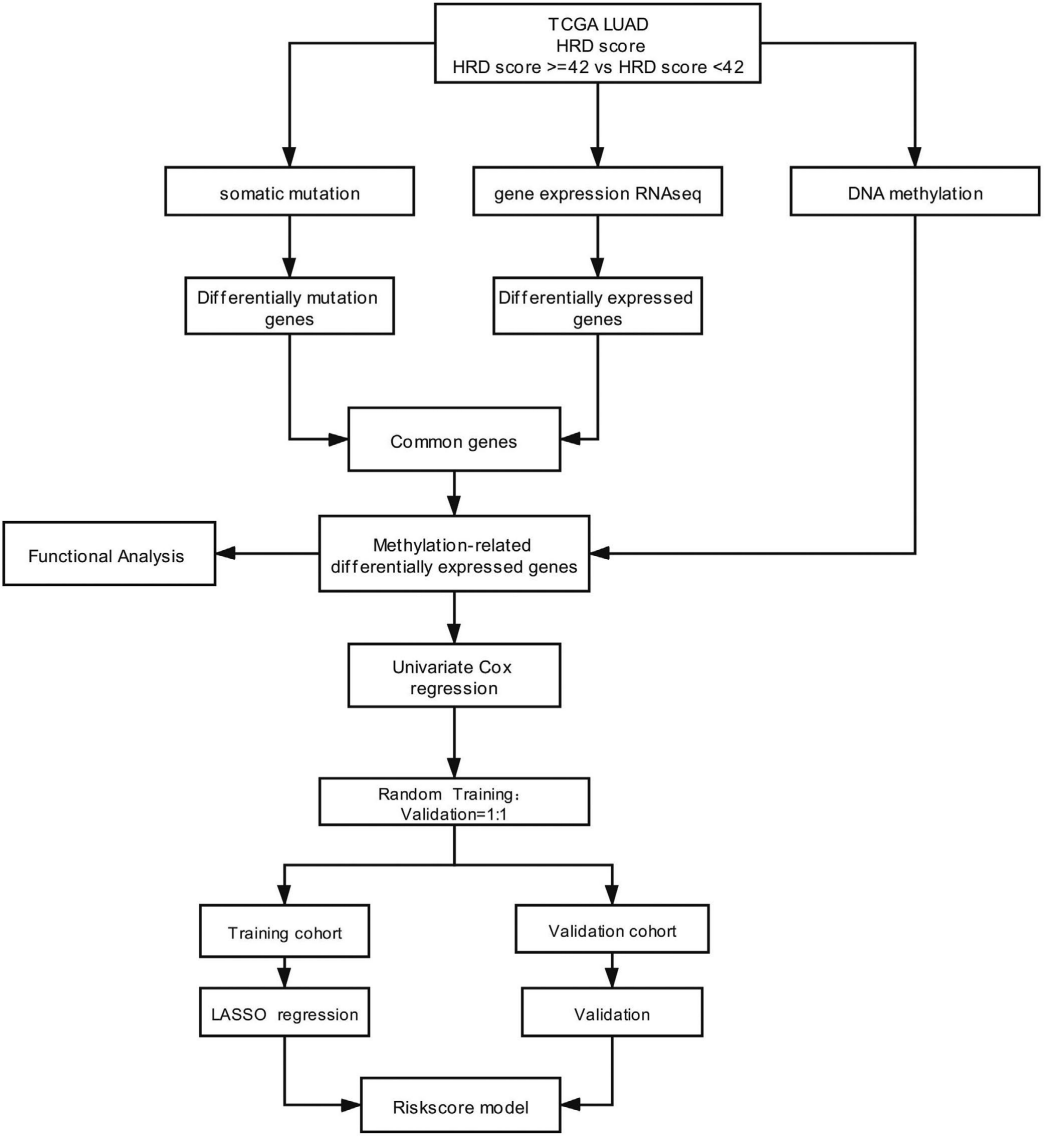
Workflow for the identification of a methylation‐associated differentially mutated and expressed gene (DMEG) signature for lung adenocarcinoma (LUAD)

In this study, we aimed to identify novel prognostic biomarkers for LUAD (Figure 1). We first investigated global HRD in LUAD. HRD‐associated differentially mutated genes (DMGs) and differentially expressed genes (DEGs) were identified to screen for HRD‐associated differentially mutated and expressed genes (DMEGs) in LUAD. We then performed DNA methylation profiling of LUAD and analyzed the DMEGs that were negatively correlated with DNA methylation. Finally, we examined the prognostic capacity of methylation‐associated DMEGs and built a powerful prognostic model for LUAD. Our study provides insights into the mechanisms underlying LUAD, highlighting the potential of methylation‐ and HRD‐related signatures for predicting clinical outcomes that may improve the clinical management of LUAD.

## 2 MATERIALS AND METHODS

### 2.1 Data collection and processing

Raw data, including RNA sequencing data, somatic mutations (single‐nucleotide polymorphisms [SNPs] and small insertion‐deletions [INDELs]), DNA methylation data, and clinical information from patients with LUAD were downloaded from The Cancer Genome Atlas (TCGA) database.^29^ A total of 250 LUAD samples with matched RNA sequencing, SNP, and DNA methylation data were used for subsequent analysis.

Ensembl_IDs were converted to Symbol_IDs using the gene mapping annotation from the Gencode database.^30^ If multiple Ensembl_IDs corresponded to the same Symbol_ID, the average values for those Ensembl_IDs were calculated and assigned to their corresponding Symbol_ID. Standardized DNA methylation data were obtained after filtering, quality control, and BMIQ normalization using the “ChAMP” package (V2.14.1) in R.^31^

### 2.2 Homologous recombination deficiency analysis

HRD was evaluated as previously described.^32^ The HRD score was calculated by examining the following: loss of heterozygosity (LOH), telomeric allelic imbalance (NtAI), and large‐scale transition (LST) based on SNP data, after which the samples were divided into high (HRD score ≥42) and low (HRD score <42) HRD score groups as previously described.^33^

Global mutations in LUAD were evaluated based on SNP and INDEL data using the “maftools” package in R.^34^ The mutation frequency in LUAD was calculated, and the top 10 mutations were visualized.

### 2.3 Analysis of DMGs

Fisher's test was performed using mafCompare function in the “maftools” package to examine the difference in mutation frequency between the high and low HRD groups, with a cutoff of *p* < 0.05.^34^ The top 10 DMGs were then visualized.

### 2.4 Analysis of the DEGs between the high and low HRD groups

A *t*‐test was performed to examine the differences in mRNA levels between the high and low HRD groups, using a cutoff of *p* < 0.05, followed by heatmap and volcano plotting of the DEGs using “ggplot2” (V3.2.1) in R.^35^ The overlapping DEGs and DMGs were considered as DMEGs.

### 2.5 Analysis of methylation‐associated DMEGs

Methylated genes were obtained by annotating the methylated sites using the FDb. InfiniumMethylation.hg19 package in R. The Pearson correlation coefficient between the mRNA levels of DMEGs and β values of methylation sites was calculated using the “cor” package in R. The cutoff value was set at *p* < 0.05 and *r* < −0.15.

### 2.6 Gene ontology (GO) and pathway enrichment analysis of the methylation‐associated DMEGs

GO and pathway enrichment analysis of the methylation‐associated DMEGs were performed using an over‐representation analysis in gProfileR, with a cutoff value of Benjamini‐Hochberg‐adjusted *p* < 0.05.^36^, ^37^, ^38^

### 2.7 Construction of a prognostic risk model

Tumor samples were randomly and equally divided into training and validation sets (125 samples each). Univariate Cox regression analysis was performed to identify the methylation‐associated DMEGs related to the overall survival of patients with LUAD. The prognosis coefficients for multiple factors were calculated using multivariate Cox regression analysis. A prognostic model was built, and risk scores were calculated using the following formula:

$$\text{Risk}\;\text{score}=\sum {\text{Coef}}_{\text{DMEG}}\times \exp_{\text{DMEG}}$$

wherein Coef_DMEG_ represents the prognostic coefficient of the DMEGs calculated using multivariate Cox regression analysis, and Exp_DEMG_ represents the expression level of the DMEGs in the training set.

### 2.8 Validation of the prognostic model

Risk scores were calculated for the training, validation, and total sets, and patients were divided into high‐ and low‐risk groups based on these scores. Kaplan‐Meier survival curves were plotted separately for the three datasets to evaluate the efficacy of the prognostic model.

### 2.9 Independence analysis of clinical features and risk score

Univariate and multivariate Cox regression analyses were performed to examine whether clinical features, such as tumor, node, metastasis (TNM) stage, age, and sex, and risk score were independent prognostic factors for LUAD. A log‐rank *p* < 0.05 was set as the cutoff value.

### 2.10 Association between clinical features and risk groups

The differences in clinical features (TNM stage, tumor stage, age, and sex) between the high‐ and low‐risk groups were evaluated using the “ggstatsplot” package (V0.5.0) in R to calculate the ratio of each clinical feature in the different risk groups, and the *p* value was calculated using the chi‐square test.^39^

## 3 RESULTS

### 3.1 HRD in LUAD

**FIGURE 2:**
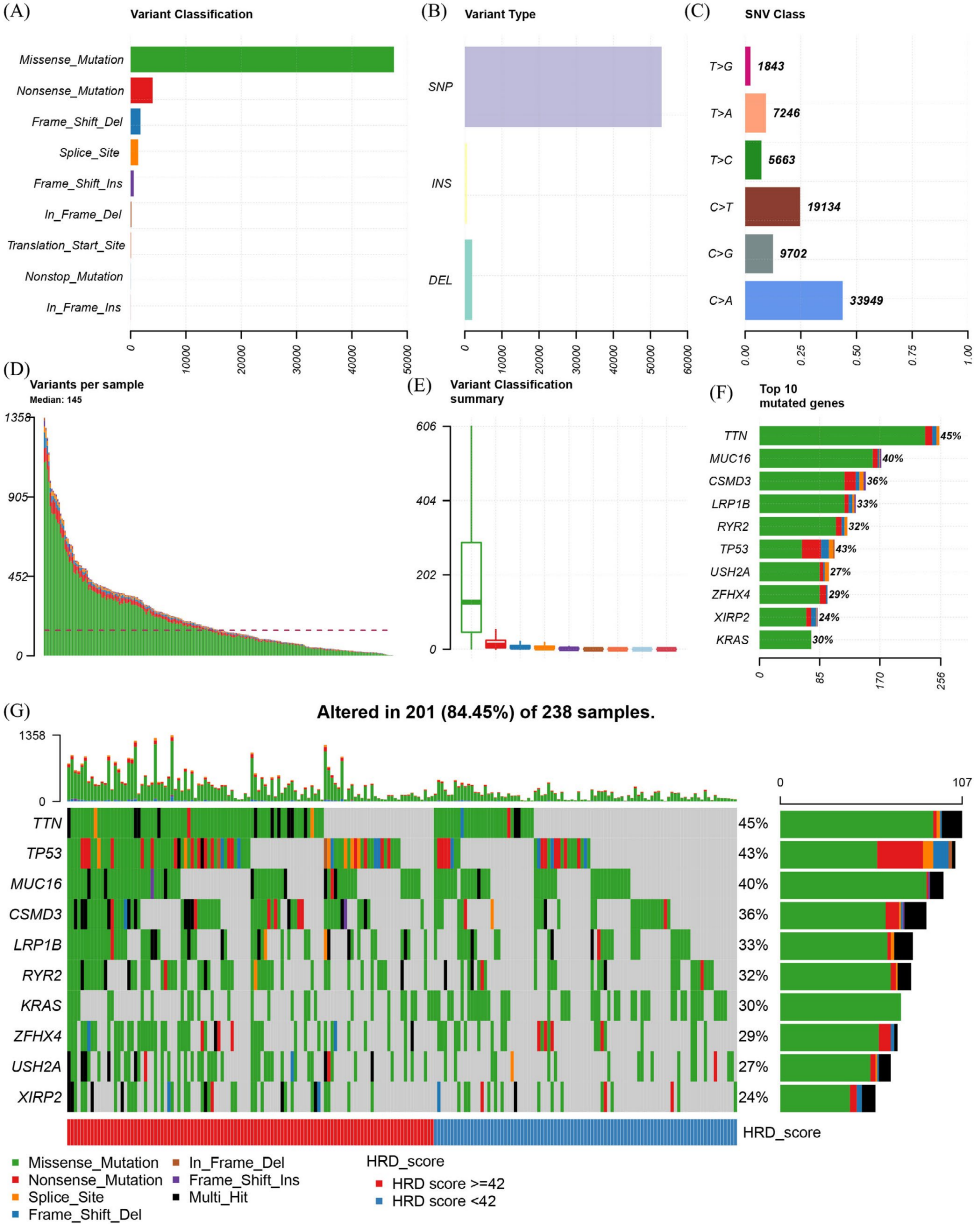
Homologous recombination deficiency in lung adenocarcinoma (LUAD). (A) Distribution of different mutation types in LUAD. (B) Distribution of single‐nucleotide polymorphisms (SNPs), insertions (INS), and deletions (DEL) in LUAD. (C) Frequency of single‐nucleotide variant (SNV) subtypes in LUAD. (D) Distribution of total mutations in individual LUAD samples. (E) Frequency of different mutation types in LUAD. (F) Top 10 most frequently mutated genes in LUAD. (G) Distribution of the top 10 most frequently mutated genes in the high and low homologous recombination deficiency (HRD) groups

Scores for the HRD subtypes NtAI, LST, and LOH as well as the total HRD score for the LUAD samples were calculated, and the patients were divided into high (HRD ≥42) and low (HRD <42) HRD score groups (Table S1). The details of the HRD patterns in LUAD are shown in Figure 1. Missense mutations were predominant in LUAD, followed by nonsense mutations (Figure 2A). SNPs were the most frequent variant type in LUAD, while INS and DEL were rare (Figure 2B). For the single‐nucleotide variants, cytosine to adenine mutations were significantly the most frequent among the mutations (Figure 2C). The number of variants in each LUAD sample was calculated, and the median number was 145 (Figure 2D). The mutation categories are shown in box plots using different colors (Figure 2E). The top 10 most frequently mutated genes were titin (*TTN*), mucin 16 (*MUC16*), CUB and Sushi multiple domains 3 (*CSMD3*), low‐density lipoprotein receptor‐related protein 1B (*LRP1B*), ryanodine receptor 2 (*RYR2*), tumor protein p53 (*TP53*), usherin (*USH2A*), zinc finger homeobox 4 (*ZFHX4*), Xin actin‐binding repeat‐containing 2 (*XIRP2*), and KRAS proto‐oncogene, GTPase (*KRAS*), as shown in Figure 2F. These genes were highly enriched in the high HRD score group (Figure 2G).

### 3.2 Classification of the methylation‐associated DMEGs between the high and low HRD score groups

**FIGURE 3:**
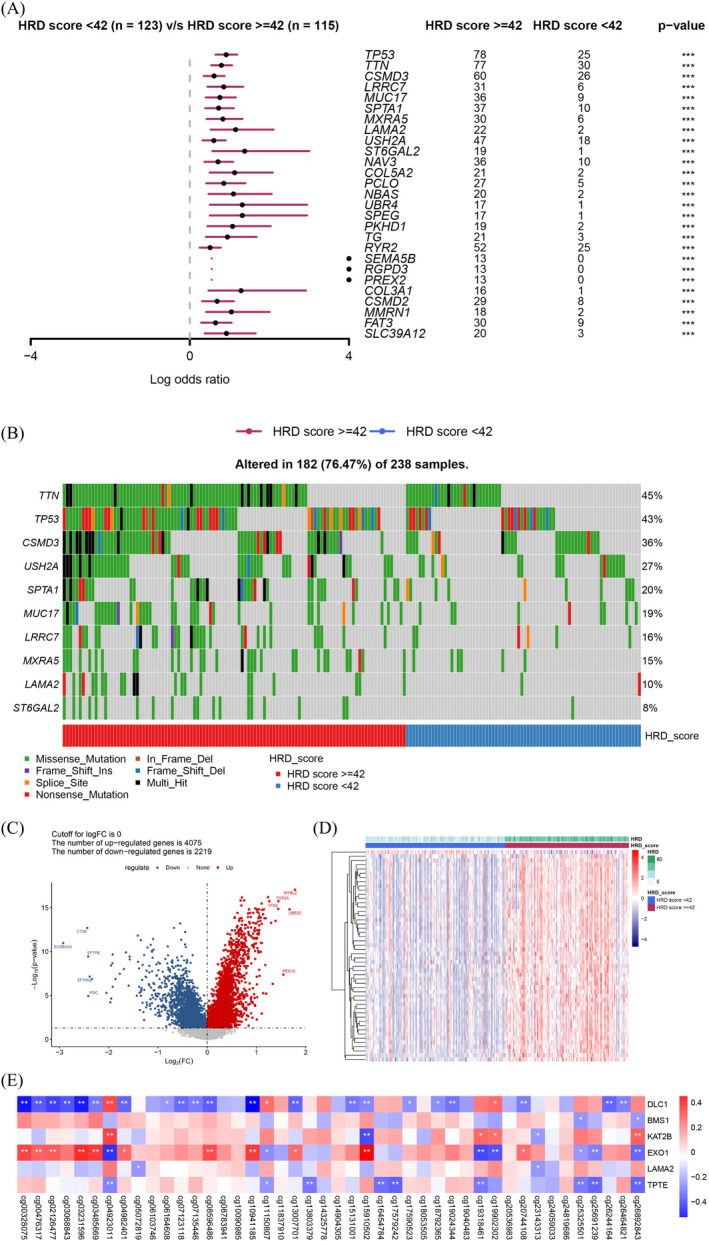
Classification of the methylation‐associated differentially mutated and expressed genes (DMEGs) between the high and low homologous recombination deficiency (HRD) score groups. (A) Differentially mutated genes (DMGs) with *p* value <0.0001 between the high and low HRD score groups. (B) Distribution of the mutations in the 10 most significant DMGs between the high and low HRD score groups. (C) Volcano plot of the DEGs between the high and low HRD score groups. Red: upregulated genes; blue: downregulated genes; and gray: non‐significant genes. The top five upregulated and downregulated genes are labeled with their gene names. (D) Heatmap of the DEGs between the high and low HRD score groups. (E) Pearson correlation between β values of the methylation sites and expression levels of the DMEGs. Representative results are shown

A total of 272 DMGs were identified (Table S2), and DMGs with a *p* value <0.0001 are shown in Figure 3A. Notably, the HRD of all these genes was significantly higher in the high HRD score group than in the low HRD score group (Figure 3A). Analysis of the distribution of the 10 most significant DMGs (*TTN*, *TP53*, *CSMD3*, *USH2A*, *SPTA1*, *MUC17*, *LRRC7*, *MXRA5*, *LAMA2*, and *ST6GAL2*) revealed more HRD in the high HRD score group than in the low HRD score group (Figure 3B).

Comparison of gene expression between the high and low HRD score groups revealed 6294 DEGs, 4075 of which were upregulated and 2219 were downregulated (Table S3 and Figure 3C,D). The top five upregulated genes were *MYBL2*, *TOP2A*, *TPX2*, *UBE2C*, and *PEG10*, while the top five downregulated genes were *CTSE*, *SCGB3A2*, *SFTPB*, *SFTPA2*, and *PGC*.

The overlapping genes between the DMGs and DEGs were considered as the DMEGs. In the LUAD samples, 57 DMEGs corresponding to 1402 methylation sites were found. Pearson correlation analysis revealed 42 genes that were significantly correlated with 259 methylation sites (Table S4). Some of the methylation‐associated DMEGs were deleted in liver cancer 1 Rho GTPase‐activating protein (*DLC1*), BMS1 ribosome biogenesis factor (*BMS1*), lysine acetyltransferase 2 B (*KAT2B*), exonuclease 1 (*EXO1*), laminin subunit alpha 2 (*LAMA2*), and transmembrane phosphatase with tensin homology (*TPTE*), as shown in Figure 3E.

### 3.3 GO and pathway enrichment analysis of methylation‐associated DMEGs

**FIGURE 4:**
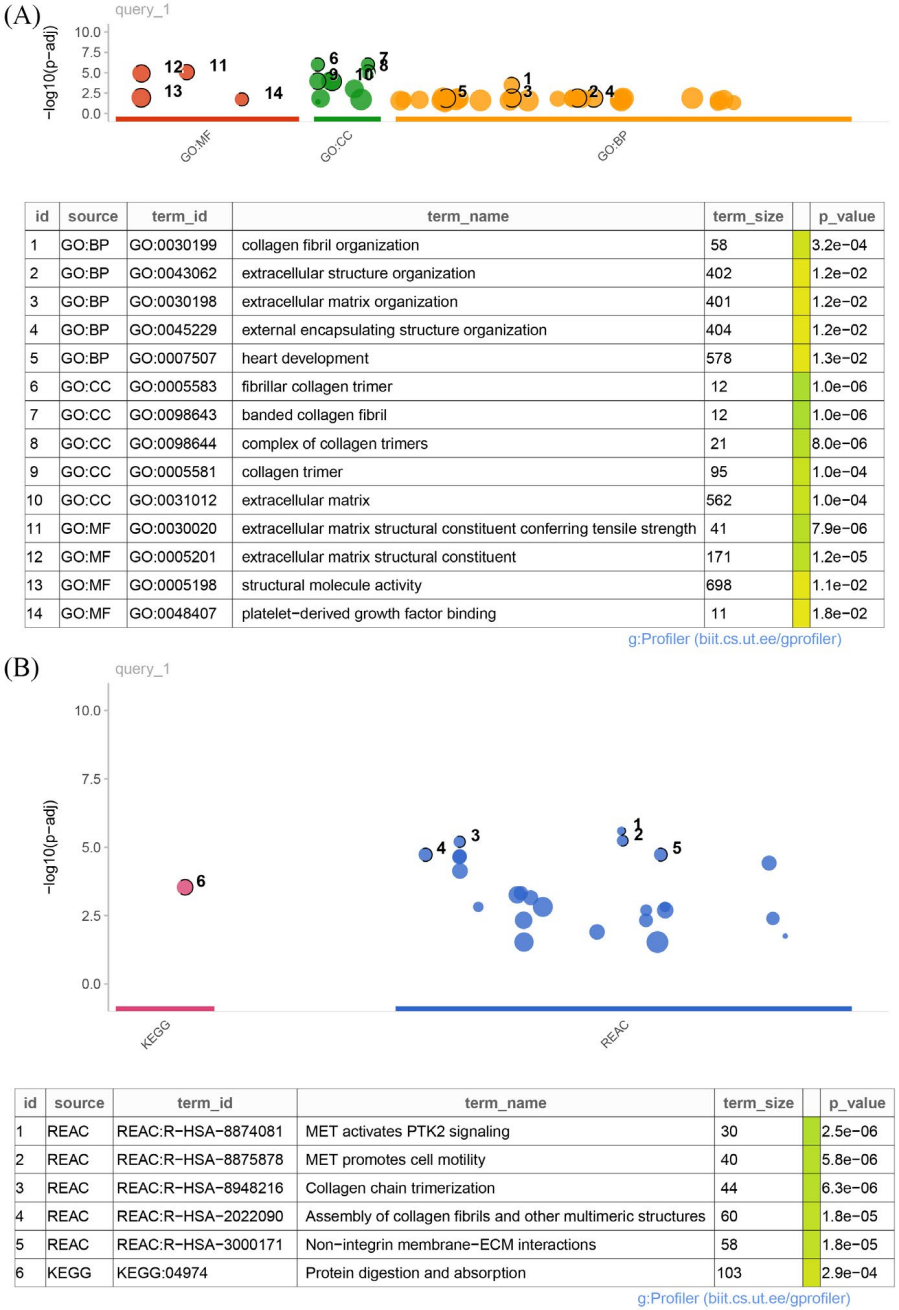
Gene ontology (GO) and pathway enrichment analysis of the methylation‐associated differentially mutated and expressed genes (DMEGs). (A) GO analysis of the DMEGs (B) Kyoto Encyclopedia of Genes and Genomes and Reactome pathway enrichment analyses of the DMEGs

GO analysis was performed to examine the features of the methylation‐associated DMEGs. The enriched GO terms included 32 biological processes, 12 cellular components, and 4 molecular functions (Figure 4A). ECM‐related components were dominant in the GO analysis. Meanwhile, KEGG and Reactome pathway analyses revealed 1 and 24 enriched signaling pathways, respectively (Figure 4B). Consistent with the results of GO analysis, ECM‐related pathways were enriched, as were mesenchymal‐to‐epithelial transition and protein digestion and absorption pathways.

### 3.4 Construction and evaluation of the prognostic model

**FIGURE 5:**
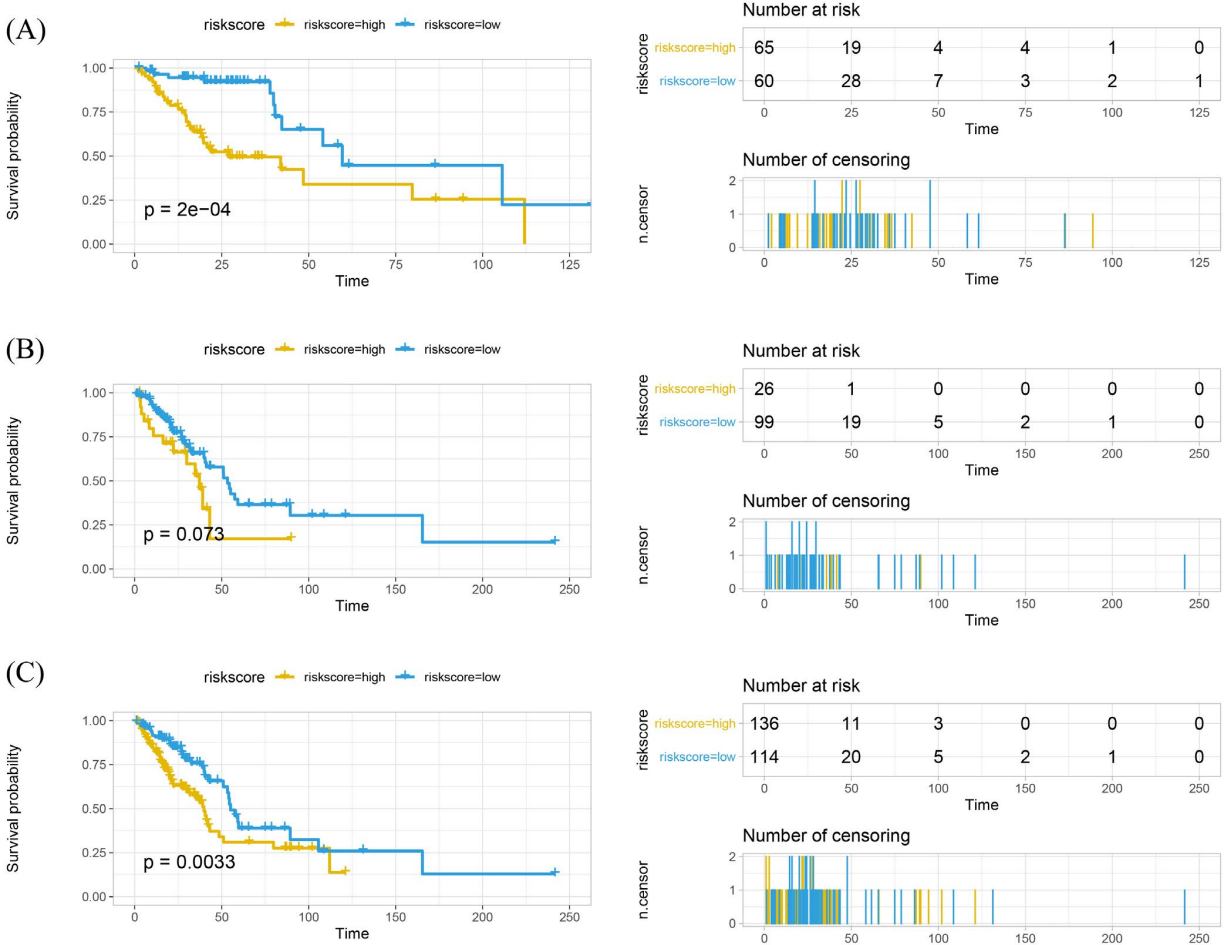
Construction and evaluation of the prognostic model. (A) Kaplan‐Meier survival curve of patients with LUAD in the training set. (B) Kaplan‐Meier survival curve of patients with LUAD in the validation set. (C) Kaplan‐Meier survival curve of all the patients with LUAD (total set)

Univariate Cox analysis of 42 methylation‐associated DMEGs was performed in the training set, and six were found to be significantly correlated with the prognosis of LUAD (Table 1). *EXO1*, *TPTE*, and *BMS1* were unfavorable factors that were correlated with worse prognosis in patients with LUAD, while *DLC1*, *KAT2B*, and *LAMA2* were favorable factors. According to their risk scores, patients in the training, validation, and total sets were divided into high‐risk (risk score ≥ median_risk score_) and low‐risk (risk score < median_risk score_) groups. Kaplan‐Meier survival curves were plotted for each set. In the training set, patients with LUAD in the high‐risk group showed significantly shorter overall survival than those in the low‐risk group (Figure 5A). The results were consistent in both the validation and total sets (Figure 5B,C). The results demonstrate that a prognostic model based on methylation‐associated DMEGs can effectively assess risk for patients with LUAD and can be used to stratify them into high‐ and low‐risk groups.

**TABLE 1:** Univariate Cox analysis of methylation‐associated DMEGs

Symbol	*p* value	HR (95% CI for HR)
EXO1	0.002384	1.61 (1.18–2.19)
DLC1	0.004676	0.683 (0.524–0.889)
TPTE	0.008129	18.6 (2.14–162)
KAT2B	0.010817	0.507 (0.301–0.855)
BMS1	0.025282	2.37 (1.11–5.06)
LAMA2	0.025301	0.653 (0.45–0.949)

### 3.5 Identification of independent prognostic factors

The independence of various factors (clinical features and risk score) was evaluated via univariate and multivariate Cox regression analyses. Among the tested parameters (TNM stage, risk score, sex, and age), only the risk score was significant in both the univariate and multivariate Cox regression analyses (Table 2 and Figure S1).

**TABLE 2:** Univariate and multivariate Cox analysis of clinical features and risk score

Clinical characteristics	Univariables cox		Multivariables cox	
	*p* value	HR (95% CI for HR)	*p* value	HR (95% CI for HR)
pathologic_N	6.117E−05	1.79 (1.35–2.38)	0.134	1.16 (0.954–1.42)
RiskGroup	0.0068033	0.516 (0.319–0.833)	0.00396	0.522 (0.335–0.812)
pathologic_T	0.0420816	1.34 (1.01–1.78)		
pathologic_M	0.1591702	1.7 (0.812–3.56)		
Gender	0.1927828	1.37 (0.853–2.2)		
Age	0.2974967	1.31 (0.787–2.18)		

### 3.6 Association between clinical features and risk group

**FIGURE 6:**
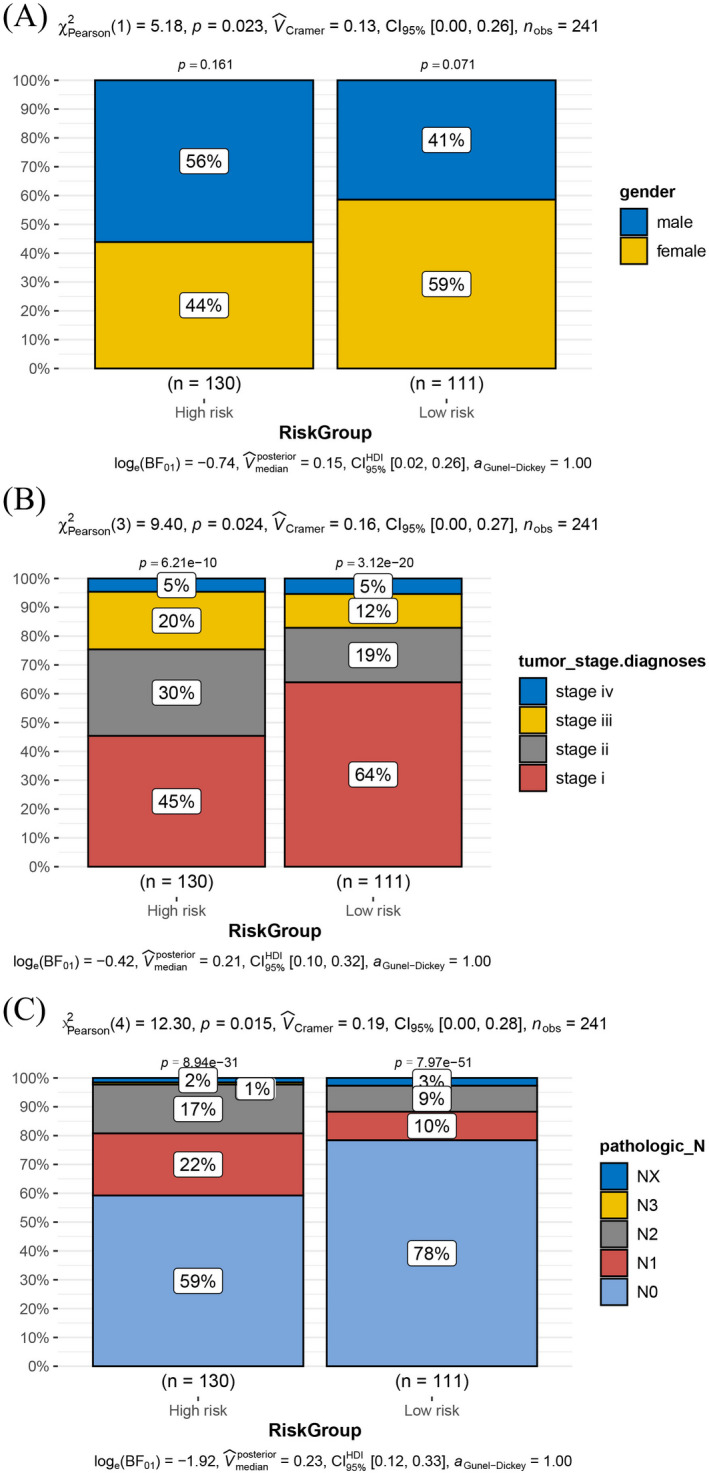
Association between clinical features and risk groups. (A) Sex distribution in the high‐ and low‐risk groups. (B) Distribution of tumor stages in the high‐ and low‐risk groups. (C) Distribution of pathologic_N stage in the high‐ and low‐risk groups

The distribution of clinical features (TNM stage, tumor stage, age, and sex) in the high‐ and low‐risk groups was examined. The high‐risk group contained more male patients, while the low‐risk group contained more female patients (Figure 6A). The tumor stage distribution was also significantly different between the high‐ and low‐risk groups. The high‐risk group included more patients with advanced LUAD, while the low‐risk group was dominated by patients with early‐stage disease (Figure 6B). Furthermore, the pathologic_N values were different between the two risk groups. Approximately 40% of patients in the high‐risk group had tumor cells in nearby lymph nodes compared with 30% of patients in the low‐risk group (Figure 6C). Other clinical features did not differ significantly between the high‐ and low‐risk groups (Figure S2A‐C).

## 4 DISCUSSION

Molecular profiling of cancers has revealed heterogeneity in tumors and holds promise for risk stratification in cancer management. Our current study identified a novel methylation‐associated HRD‐related signature that can predict the prognosis of patients with LUAD. HRD is common in LUAD, and patients with high HRD show different mutation patterns compared to those with low HRD. The differentially expressed HRD‐related mutant genes in LUAD were associated with DNA methylation. DNA‐associated DMEGs can be used to effectively stratify patients into high‐ and low‐risk groups, and the overall survival of patients in the high‐risk group was significantly shorter than that of patients in the low‐risk group. Further investigation of the association of the prognostic model with clinical features revealed that the high‐risk group included patients with more advanced disease and with lymph node metastasis. Further studies are needed to explore the relationship between methylation‐associated DMEGs and the carcinogenesis of LUAD.

HRD is a common feature of cancers,^13^, ^14^ and comprehensive studies of HRD have shown its potential in predicting cancer prognosis.^15^ Although HRD has been previously reported in LUAD, its prognostic value remains to be evaluated.^15^, ^16^ Together with methylation profiling, we developed a methylation‐ and HRD‐based molecular signature that showed good separation of high‐ and low‐risk patients. More importantly, the risk score was an independent prognostic factor for LUAD. This prognostic model exhibits such strong prognostic power as it includes more patients with advanced stage disease and with lymph node metastasis in the high‐risk group.

HRD results in the failure of DNA mismatch repair and leads to DNA mutations. The extent of HRD is positively correlated with DNA mutations; in LUAD, patients with higher HRD had more DNA mutations. A recent study found that mutations in *TTN* were an indicator of tumor mutation burden in multiple cancers,^40^ which implies that mutations are correlated with HRD. This is consistent with our finding that *TTN* had the highest mutation rate and was enriched in the high HRD score group. Dysregulated methylation is another driver of carcinogenesis,^20^, ^21^, ^22^, ^23^ and the combination of methylation and gene mutations resulted in a good prognostic biomarker for LUAD. *DLC1* was negatively associated with prognosis in LUAD, with a hazard ratio of 0.68, which is consistent with studies showing its suppressive activity in LUAD cells,^41^, ^42^ Expression of *KAT2B* is decreased in many cancers, and *KAT2B* can regulate *SHC3*, an immune‐related gene that is associated with the prognosis of LUAD.^43^, ^44^ *EXO1* is a prognostic biomarker for LUAD and is associated with immune cell infiltration.^45^ Our study revealed the relationship between HRD and methylation, further validating its prognostic role in LUAD. The role of *TPTE* in LUAD has not been reported; this study is the first to report its association with LUAD prognosis, with an extremely high hazard ratio. It may be interesting to investigate the role of *TPTE* in LUAD in future studies. Most methylation‐associated genes with mutations showed a strong association with progression, suggesting that the methylation‐HRD signature is involved in carcinogenesis and their prognosis‐predicting ability.

In this study, we identified 42 methylation‐associated DMEGs that were related to ECM components, ECM remodeling, and signaling. Epigenetic regulation of ECM remodeling plays an important role in carcinogenesis.^46^, ^47^ In nonsmall cell lung carcinoma, DNA methylation and other epigenetic modifications of ECM remodeling genes are associated with epithelial‐mesenchymal transition.^48^ However, the roles of methylation‐associated ECM‐related genes in LUAD have not yet been reported. Our study is the first to report the methylation of ECM‐related genes in LUAD and suggests their association with LUAD prognosis: *DLC1* participates in cytoskeleton organization, while *LAMA2* is an ECM component.^49^, ^50^ This evidence suggests that the methylation of ECM‐related pathways is important and may be relevant to the malignancy of LUAD.

However, there are some limitations to the current study. Although we set up an independent validation set for our prognostic model, it was still within the same cohort as the training set. Therefore, multiple independent cohorts are required to validate our findings. In addition, while the model exhibited powerful prognostic capacity, and the genes used for its construction were associated with LUAD, further biological studies are required to investigate their roles in LUAD progression and verify our prognostic model.

In summary, the present study profiled the HRD‐related gene mutations in LUAD and developed a methylation‐associated DMEG signature for predicting the overall survival of patients with LUAD. Our findings provide a better understanding of LUAD and highlight the potential of methylation‐ and HRD‐related signatures for clinical outcome prediction.

## CONFLICT OF INTEREST

The authors declare that they have no conflict of interest.

## AUTHOR CONTRIBUTIONS

Conception and design of the research: WY; data acquisition: JL; data analysis and interpretation: YC and YS; statistical analysis: SZ; drafting of the manuscript: GN; manuscript revision for important intellectual content: AZ and JK‐L. All authors read and approved the final manuscript.

## Supporting information

Fig S1

Fig S2

Table S1

Table S2

Table S3

Table S4

Supplementary Material

## Data Availability

The data that support the findings of this study are openly available in The Cancer Genome Atlas (TCGA; http://cancergenome.nih.gov/) and Gencode (https://www.gencodegenes.org/) databases.
