# Supplementary figures and images for: Methylation‐ and homologous recombination deficiency‐related mutant genes predict the prognosis of lung adenocarcinoma
Source: J Clin Lab Anal. 2022 Mar 3;36(4):e24277. doi: 10.1002/jcla.24277 (PMC8993616; doi:10.1002/jcla.24277)

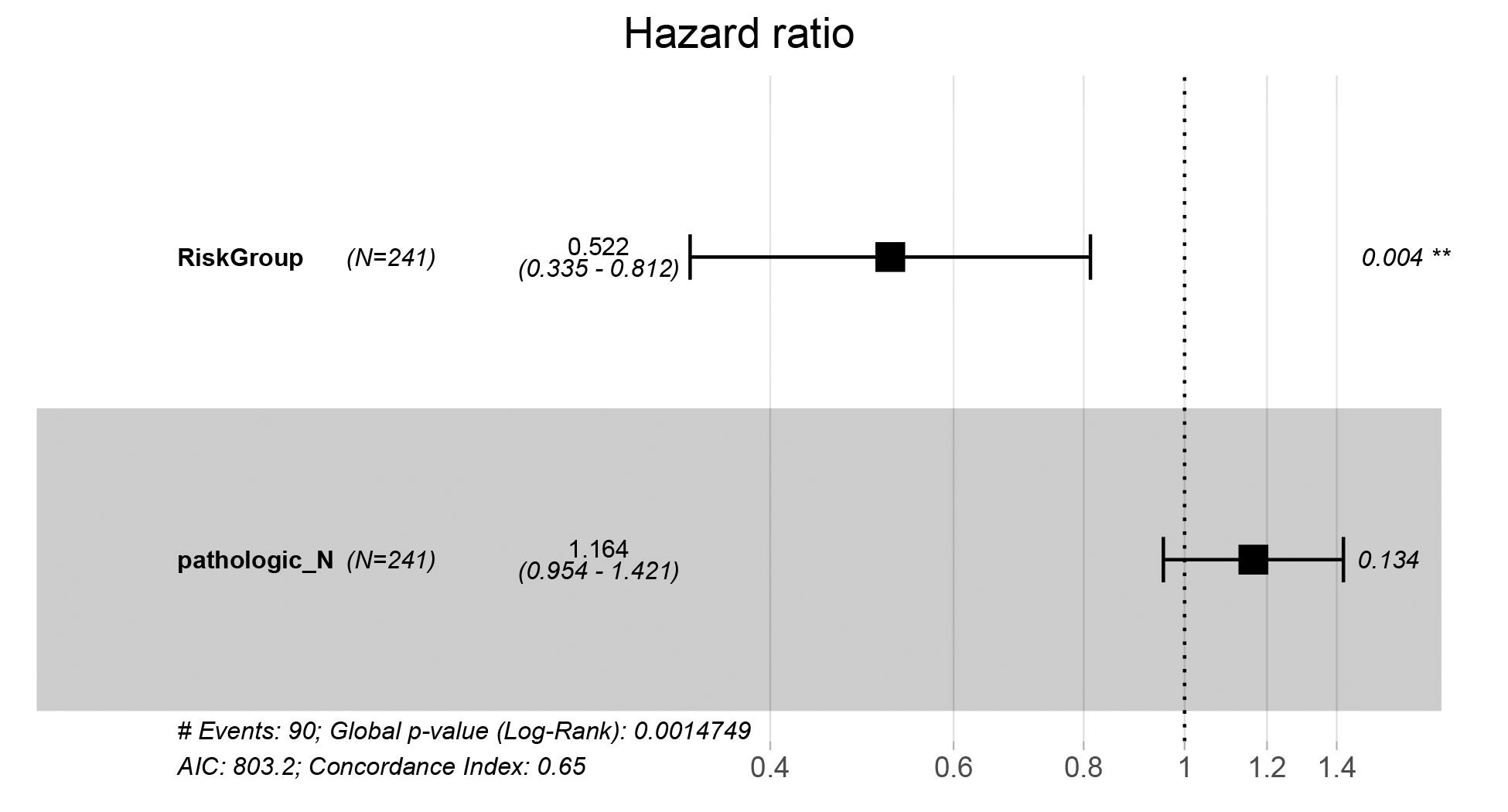

Supplement: Supplementary file 1 — Fig S1 [file JCLA-36-e24277-s006.jpg]

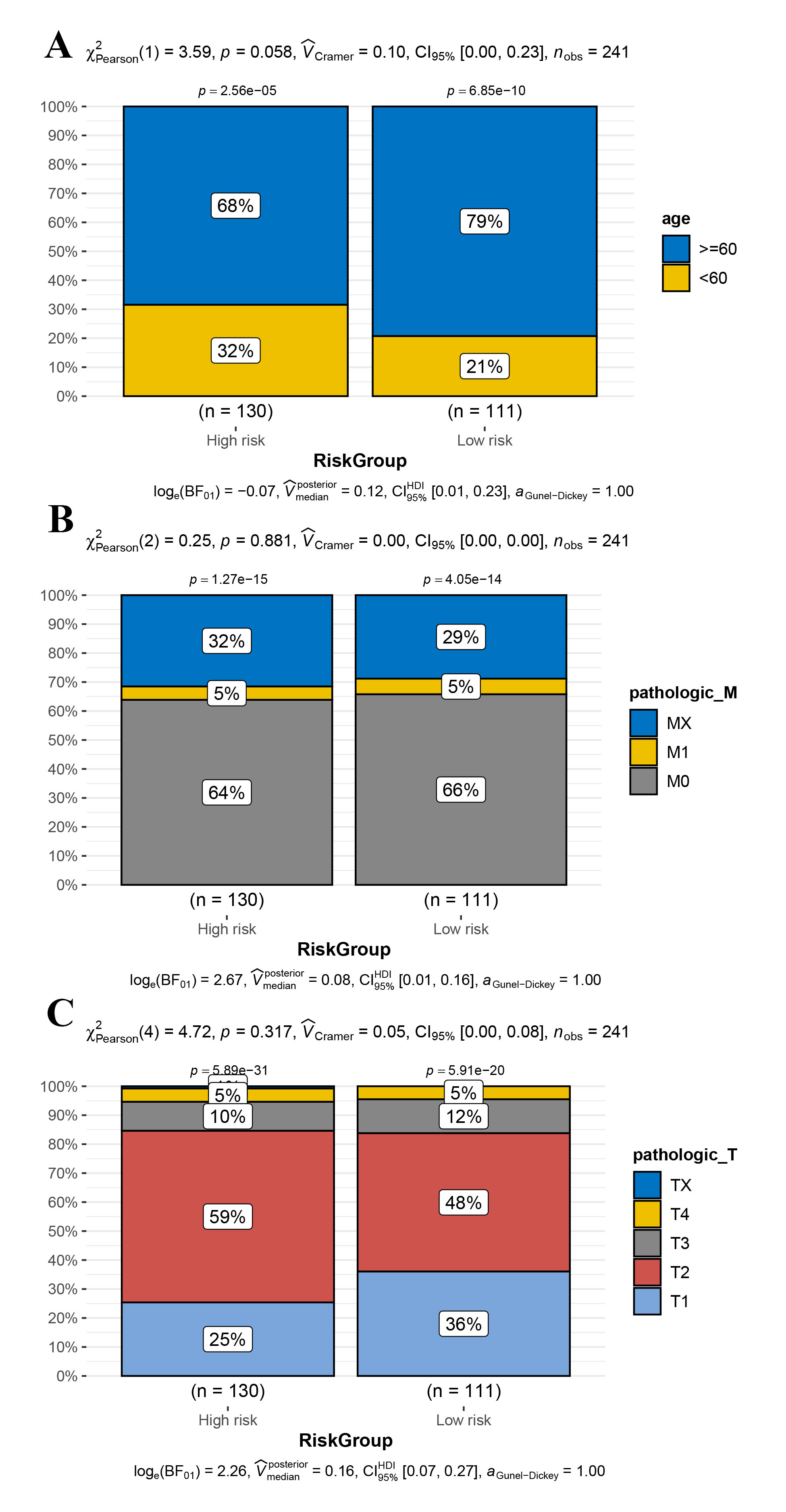

Supplement: Supplementary file 2 — Fig S2 [file JCLA-36-e24277-s004.jpg]
