# Supplementary material for: Methylation‐ and homologous recombination deficiency‐related mutant genes predict the prognosis of lung adenocarcinoma
Source: J Clin Lab Anal. 2022 Mar 3;36(4):e24277. doi: 10.1002/jcla.24277 (PMC8993616; doi:10.1002/jcla.24277)
Supplement: Supplementary file 7 — Supplementary Material [file JCLA-36-e24277-s001.docx]

**Supplementary materials**

**Supplementary Figure S1. Identification of independent prognostic factors for lung adenocarcinoma (LUAD). (A-C**) Visualization of multivariate Cox regression analysis.

**Supplementary Figure S2. Association between clinical features and risk groups. (A)** Age distribution in the high- and low-risk groups. (**B)** Distribution of pathologic_M stage in the high- and low-risk groups. (**C)** Distribution of pathologic_T stage in the high- and low-risk groups.

**Supplementary Table 1.** Homologous recombination deficiency (HRD) scores in lung adenocarcinoma (LUAD).

**Supplementary Table 2.** Differentially mutated genes in the high and low homologous recombination deficiency (HRD) score groups.

**Supplementary Table 3.** Differentially expressed genes in the high and low homologous recombination deficiency (HRD) score groups.

**Supplementary Table 4.** Pearson correlation between β values of methylation sites and expression levels of differentially mutated and expressed genes (DMEGs).
